# A new framework for tailoring laparoscopic cholecystectomy: Integrating preoperative clinical factors with surgical difficulty based on the Tokyo Guidelines 2018

**DOI:** 10.1002/jhbp.12145

**Published:** 2025-04-10

**Authors:** Daisuke Noguchi, Aoi Hayasaki, Takahiro Ito, Yusuke Iizawa, Takehiro Fujii, Akihiro Tanemura, Yasuhiro Murata, Naohisa Kuriyama, Masashi Kishiwada, Shugo Mizuno

**Affiliations:** ^1^ Department of Hepatobiliary Pancreatic and Transplant Surgery Mie University Graduate School of Medicine Tsu Mie Japan

**Keywords:** acute cholecystitis, preoperative prediction, subtotal cholecystectomy, surgical difficulty score, Tokyo guideline 2018

## Abstract

**Purpose:**

The Tokyo Guidelines 2018 introduced the Surgical Difficulty Score (TGDS18) to assess laparoscopic cholecystectomy (LC) difficulty based on intraoperative findings. This study aimed to predict surgical difficulty preoperatively using clinical factors correlated with TGDS18.

**Methods:**

Of 369 LC cases for cholecystitis (Jan 2014–Jul 2024), 106 with operative video data were analyzed. Multivariate analysis of 69 with preoperative CT (≤14 days) evaluated the association between preoperative clinical findings and TGDS18 sub‐scores (around the gallbladder, Calot's triangle, gallbladder bed, additional findings, unrelated to inflammation).

**Results:**

TGDS18 was positively correlated with operative time, blood loss, and hospital stay (all *p* < .001). Patients undergoing subtotal cholecystectomy had higher TGDS18 scores (median 20, *p* < .001). Six preoperative findings strongly associated with TGDS18 sub‐scores were identified: calcified stone in cystic duct, TG18 Grade ≥2, preoperative gallbladder drainage, urgent operation, pericholecystic inflammation, and age‐adjusted Charlson comorbidity index ≥7. The rate of subtotal cholecystectomy increased with the number of findings linked to the “Calot's triangle” sub‐score—cystic duct stone and TG18 Grade ≥2. (0% with no findings, 8% with one finding, and 23% with both, *p* = .009). Similarly, the risk of cholecystectomy requiring the posterior wall left can be predicted by the number of clinical findings related to the ‘Gallbladder bed’ sub‐score (*p* = .009).

**Conclusions:**

The clinical findings linked to TGDS18 allow tailored preoperative strategies for acute cholecystitis.

## Graphical abstract

Noguchi and colleagues at Mie University first identified preoperative clinical findings linked to the Surgical Difficulty Score from the Tokyo Guidelines 2018 for laparoscopic cholecystectomy. These findings enable preoperative assessment of local surgical difficulty at sites like Calot's triangle or the gallbladder bed, facilitating the prediction of high‐difficulty cases, including those requiring subtotal cholecystectomy.

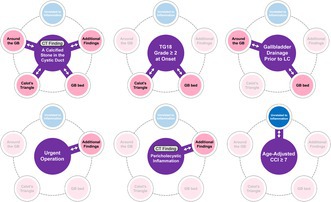

## 1 INTRODUCTION

The Tokyo Guidelines 2018 (TG18) introduced the Surgical Difficulty Score (TGDS18) to universally and objectively evaluate the difficulty of laparoscopic cholecystectomy (LC). This scoring system was developed by achieving consensus among LC experts from Japan, South Korea and Taiwan through the Delphi method.^1^ TGDS18 successfully quantifies surgical difficulty by combining 25 intraoperative findings known to contribute to the complexity of LC. Historically, studies evaluating LC difficulty have used markers such as operative time^2^, ^3^, ^4^ and conversion rates to open surgery^5^, ^6^, ^7^, ^8^, ^9^ as proxies for surgical difficulty. However, there has been debate on whether these surrogate markers truly reflect objective difficulty, as they are significantly influenced by factors such as the surgeon's skill and institutional policies.

Before TGDS18, the Nassar scale,^10^ developed in 1995, was one of the few systems to directly assess surgical difficulty by intraoperative findings, and its utility was multiply validated in large cohorts.^11^, ^12^, ^13^, ^14^ However, the Nassar scale has some limitations. It inadequately evaluates inflammatory findings affecting the difficulty due to omitting factors such as hemorrhagic and edematous changes. Factors unrelated to inflammation such as severe visceral fat or liver cirrhosis, which contribute to surgical difficulty, have not been assessed. Acute cholecystitis is uniformly classified as Grade 3 (moderate) regardless of severity, which is not helpful for managing LC in cases of acute cholecystitis.^15^ Moreover, the Nassar scale lacks clear organization of its subcategories within the three major items—gallbladder, cystic pedicle, and surrounding tissue adhesions—resulting in the conflation of inflammatory findings, such as fibrosis and scarring, with non‐inflammatory findings, such as anatomical abnormalities. In contrast, TGDS18 overcomes these issues by clearly separating inflammatory from non‐inflammatory findings. Incorporating the degree and extent of fibrosis and scarring to assess localized inflammation, along with evaluating critical local anatomy for LC—including pericholecystic structures, Calot's triangle, and the gallbladder bed—as individual sub‐scores distinguishes TGDS18 from previous models and provides valuable utility preferred by clinicians.

The difficulty of LC varies significantly depending on the case,^16^ and preoperative evaluation of LC's difficulty is considered challenging. While TGDS18 is an excellent tool for intraoperative assessment, since it is constructed based on intraoperative findings, it cannot be used for preoperative assessment. Early surgery is the gold standard for acute cholecystitis^17^ and a gateway operation, where junior surgeons sometimes face unexpectedly challenging cases, causing complications such as bile duct injury (BDI). By analyzing the correlation between preoperative clinical factors and the difficulty score, the present study aimed to establish a new framework for predicting surgical difficulty and treatment strategies based on TGDS18 before operation.

## 2 METHODS

### 2.1 Patient selection and perioperative clinical findings

From January 2014 to July 2024 at our institution, 369 patients underwent LC for cholecystitis. Two hundred and sixty‐three cases without operative video data were excluded, and the remaining 106 cases were included in the present cohort. To analyze the relationship between clinical findings and TGDS18, of the 106 cases, 69 cases with CT images within 14 days prior to LC were assessed (Figure S1). This study was approved by the institutional ethics committee of Mie University hospital with approval number H2023‐227.

As shown in Table 1, we analyzed perioperative patient demographics, including age, gender, symptoms, cholecystitis severity, operative findings, TGDS18, and postoperative outcomes. Given that LC is sometimes performed electively, some variables were defined separately for the timing of onset and the closest date before surgery. In analyzing the associations between TGDS18 and preoperative findings, we selected variables for their objectivity and feasibility of collection across facilities. Preoperative imaging findings were based on plain CT scans performed within 14 days before surgery. For objectivity, findings requiring contrast‐enhanced imaging (e.g., gallbladder wall edema, thickening, necrosis) or those subject to interpretative variability (e.g., adhesions) were excluded. We finally selected calcified (radiopaque) cystic duct stones, as well as pericholecystic fluid and inflammation (Figure S2). The age‐adjusted Charlson comorbidity index (aa‐CCI) was calculated for each patient based on the original study^18^ and used as a comprehensive measure of comorbidities. Patients were screened for organ failure (heart, pulmonary, renal, or liver), coronary artery and peripheral vascular disease, cerebrovascular disease, dementia, connective tissue diseases, ulcers, diabetes, hemiplegia, solid or hematological malignancy, and AIDS/HIV, with additional points for age adjustment.

**TABLE 1:** Patient demographics and perioperative results.

Variables	Cohort with video data (*n* = 106)	Cohort with both video data and CT imaging (*n* = 69)	*p‐*Value
Age (y.o.)	67 [75–52]	70 [59–75]	.375
Gender: Male/female (%)	58 (55)/48 (45)	46 (67)/23 (33)	.116
BMI (kg/m^2^)	24 [22–27]	24 [22–27]	.686
Age adjusted Charlson comorbidity index	4 [2–6]	4 [3–6]	.424
Previous abdominal operation (%)	33 (31)	24 (35)	.615
Interval between onset and LC (day)	36 [14–67]	29 [8–45]	.066
Symptoms at onset (%)			
High fever ≥38°	26 (25)	21 (30)	.389
Murphy's sign	34 (32)	25 (36)	.570
Cholecystitis severity at onset (%)			
TG18 Grade 1/2/3	70 (66)/33 (31)/3 (3)	41 (59)/26 (38)/2 (3)	.663
Pericholecystic or liver abscess	15 (14)	13 (19)	.408
Mirizzi syndrome	2 (2)	2 (3)	.647
Blood test at onset			
WBC (/μL)	7710 [5530–11 450]	8295 [6000–13 570]	.538
CRP (mg/dL)	0.8 [0.1–8.8]	2.2 [0.2–12.2]	.157
Intervention before operation (%)			
Gallbladder drainage	14 (13)	13 (19)	.313
Endoscopic lithotomy	31 (29)	18 (26)	.649
Recent preoperative blood test			
WBC (/μL)	6120 [4850–7870]	6000 [4520–8730]	.835
CRP (mg/dL)	0.2 [0.1–0.9]	0.4 [0.1–2.1]	.057
Operative findings			
Urgent operation (%)	16 (15)	13 (19)	.515
Operative time (min)	117 [82–177]	127 [88–195]	.250
Blood loss (g)	0 [0–15]	1 [0–50]	.529
Open convert (%)	6 (6)	4 (6)	1.000
Reason for open convert			
Severe inflammation in Calot's triangle	3	2	
Vascular injury	1	1	
Bile duct injury	1	1	
Extensive adhesions by previous operation	1	0	
Subtotal cholecystectomy (%)^a^	7 (7)	5 (7)	1.000
Biliary duct injury (%)	1 (1)	1 (1)	1.000
Vascular injury (%)	1 (1)	1 (1)	1.000
Surgical difficulty score (TGDS18)			
Total	6 [3–13]	9 [3–14]	.242
Sub‐score			
Around the gallbladder	2 [0–2]	2 [0–2]	.640
Calot's triangle	2 [0–3]	2 [0–4]	.264
Gallbladder bed	1 [0–3]	2 [0–4]	.213
Additional findings of the gallbladder and its surroundings	1 [0–4]	1 [0–4]	.170
Unrelated to inflammatory findings	0 [0–2]	0 [0–1]	.544
Postoperative course			
Length of stay in hospital (day)	4 [3–7]	5 [3–10]	.154
Postoperative complication (%)			
Clavien‐Dindo ≥Grade 3a	4 (4)	4 (6)	.714
Sepsis	1 (1)	1 (1)	1.000
Bile leakage	2 (2)	2 (3)	.647
Intraabdominal abscess	2 (2)	2 (3)	.647

Abbreviations: BMI, body mass index; CRP, c‐reactive protein; TG18, Tokyo Guidelines 2018; WBC, white blood cell.

^a^ Subtotal cholecystectomy was defined as partial cholecystectomy with leaving the gallbladder neck in this study.

### 2.2 Therapeutic strategy for patients with cholecystitis and surgical procedure

Based on TG18 grading and the management flowchart,^19^ we aim to perform early cholecystectomy whenever feasible in cases deemed suitable for urgent surgery. For patients who cannot tolerate surgery or present with severe symptoms, including shock status, we avoid surgery and instead select alternative treatments such as gallbladder drainage. Endoscopic gallbladder drainage was not performed in this study. For patients with mild symptoms who recover with conservative therapy alone, elective LC is typically performed due to limited surgical and anesthesia staff for emergencies.

In our institution, a board‐certified surgeon (hepato‐biliary‐pancreatic or endoscopic Surgery) always scrubs in LC to ensure safety, aiming for a critical view of safety (CVS) according to safe steps in TG18.^20^ For cases with hardly obtaining CVS, bailout procedures like subtotal cholecystectomy or open conversion are adopted to prevent vasculo‐biliary injury. This judgment, bailout or not, was made intraoperatively and subjectively by surgeons in each LC. For all cases, especially those with suspected cystic duct stones, we confirmed the absence of residual stones intraoperatively using methods like cholangiography, stone identification in the specimen, and bile drainage verification. No postoperative residual cystic duct stones were found in this study.

### 2.3 Definition of subtotal cholecystectomy in the present study

Subtotal cholecystectomy, in a broad sense, refers to a surgical technique where part of the gallbladder wall is intentionally left behind, without specific consideration of the site or extent of the residual wall. Clinically, it can be categorized into two types: one where the wall on the neck side is preserved due to difficulty in obtaining CVS, followed by reconstitution or fenestration (neck‐preserving subtotal), and another where the posterior wall is left when detaching the gallbladder from the gallbladder bed is difficult, such as in cases of necrosis (posterior wall‐preserving subtotal). The neck‐preserving subtotal is technically more challenging and carries higher risks, including bile leakage in the early postoperative period and the potential for residual gallstones or gallbladder cancer in the long term. In this study, to avoid terminological confusion and focus on the more clinically significant issue, we defined the neck‐preserving procedure alone as ‘subtotal cholecystectomy’ in the narrow sense, while referring to the posterior wall‐preserving procedure as LC with leaving the posterior wall.

### 2.4 Surgical video evaluation based on surgical difficulty items in Tokyo Guidelines 2018

TGDS18 was retrospectively calculated with an operation video of LC by a surgeon who had a board certification. According to 25 surgical difficulty items in five categories (Figure S3): (a) appearance around the gallbladder; (b) appearance of Calot's triangle area; (c) appearance of the gallbladder bed; (d) additional findings of the gallbladder and surroundings; and (e) intra‐abdominal findings unrelated to inflammation, intraoperative findings in every case were scored, and the total difficulty score was defined as the sum of all items. In evaluating the 25 surgical difficulty items, we referred to a library of typical video clips on the website of the Japanese Society of Hepato‐Biliary‐Pancreatic Surgery (http://www.jshbps.jp/modules/project/index.php?content_id=13).

The objectivity of TGDS18 used in this study (Primary Score) was statistically validated as follows. Its agreement with the assessed score by another surgeon (Control Score) was evaluated using the intraclass correlation coefficient (ICC). Significant agreement was confirmed for all sub‐scores (ICC ≥0.8, indicating good agreement) and the total score (ICC ≥0.9, indicating excellent agreement) (Table S1).^21^ In the Bland–Altman plot for the total score, nearly all data fell within the 95% limits of agreement. The linear regression analysis showed no proportional bias (*R* ^2^ < 0.1, *p* = .417) (Figure S4).

### 2.5 Statistical analysis

Continuous variables were expressed as medians and interquartile ranges, and comparisons between two groups were made using the Mann–Whitney *U* test. Categorical variables were summarized as counts and percentages and compared using Pearson's Chi‐square test or Fisher's exact test for two groups, and the Cochran‐Armitage trend test for three or more groups. We used Spearman's rank correlation test to evaluate the correlation between TGDS18 and postoperative outcomes. To analyze associations with TGDS18, 19 preoperative clinical variables were reviewed (Table S2). For continuous clinical variables, Spearman's rank correlation was performed as a univariate analysis to assess their correlation with all sub‐scores in TGDS18 (Table S3). For categorical variables, univariate analysis was performed by comparing TGDS18 sub‐scores across categories using the Mann–Whitney *U* test (Table S4). Significant variables identified in univariate analysis were then included in a multivariate linear regression analysis to detect preoperative factors potentially associated with increased scores in each TGDS18 sub‐score. All tests were two‐sided, and a *p*‐value <.05 was considered statistically significant. All analyses were conducted using IBM SPSS Statistics version 29 (IBM Corporation, Armonk, NY).

## 3 RESULTS

### 3.1 Patient demographics and perioperative results

In 106 patients with video data, the median age was 67 years, and 55% were male. The median aa‐CCI was 4. The median time of interval between onset and LC was 36 days, and 15% of patients underwent urgent surgery. According to the TG18 severity classification, 66% were classified as Grade 1, 31% as Grade 2, and 3% as Grade 3. Two patients (2%) were diagnosed with Mirizzi syndrome with cholecysto‐choledochal fistula. Gallbladder drainage prior to LC was performed in 14 patients (13%) (Table 1). The median operative time was 117 min, and the median blood loss was 0 g. Bailout procedures included open conversion in 6 patients (6%) and subtotal cholecystectomy with reconstitution or fenestration in 7 patients (7%). The most common reason for conversion to open surgery was severe inflammation in Calot's triangle (*n* = 3). Other reasons included vascular injury, bile duct injury, and severe intra‐abdominal adhesions from prior surgery that prevented laparoscopic manipulation, each occurring in one case. One patient (1%) experienced an intraoperative bile duct injury, and one patient (1%) sustained a vascular injury. The median total TGDS18 score was 6, with sub‐scores as follows: 2 for findings around the gallbladder, 2 for Calot's triangle, 1 for the gallbladder bed, 1 for additional findings around the gallbladder, and 0 for unrelated inflammatory findings. The median hospital stay was 4 days. Postoperatively, one patient (1%) developed sepsis, two patients (2%) experienced bile leakage, and two patients (2%) had intra‐abdominal abscesses. The incidence of complications greater than Clavien‐Dindo Grade 3a was 4% (*n* = 4). No significant differences were observed in any clinical variables between the cohort with video data (*n* = 106) and those with both video data and CT imaging (*n* = 69), which was used in the analysis of the relationship with TGDS18.

### 3.2 Correlation between surgical difficulty score and perioperative outcomes

**FIGURE 1:**
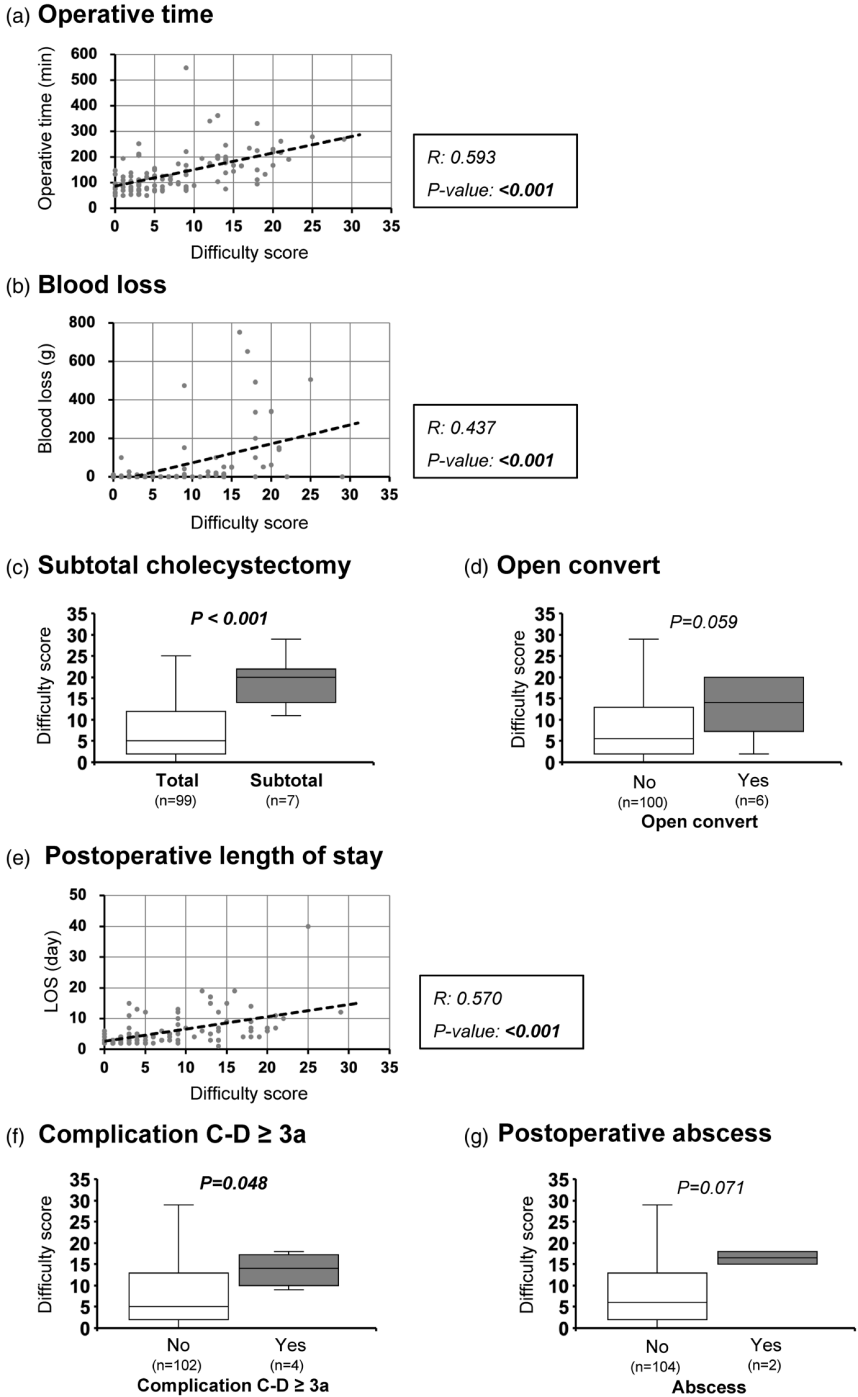
Correlation between surgical difficulty score and perioperative outcomes. The correlation between the surgical difficulty score (TGDS18) and perioperative outcomes (106 LC cases with video data were assessed). (a) Operative time (*R* = .593, *p* < .001) and (b) blood loss (*R* = .437, *p* < .001) showed a positive correlation with TGDS18. (c) Subtotal cholecystectomy cases had higher scores than cholecystectomy cases (*p* < .001). (d) Cases requiring open conversion had higher scores than cases without conversion (*p* = .059). (e) Postoperative length of hospital stay showed a positive correlation with TGDS18 (*R* = .570, *p* < .001). Scores were significantly higher in (f) cases with complications ≥Clavien‐Dindo Grade 3a (*p* = .048) and (g) cases with intra‐abdominal abscess (*p* = .071). LC, laparoscopic cholecystectomy; TGDS18, surgical difficulty score from Tokyo Guidelines 2018.

In 106 LC cases with video data, the correlation between TGDS18 and perioperative outcomes was evaluated. As scores increased, operative time was significantly longer (*R* = 0.593, *p* < .001, Figure 1a) and blood loss was greater (*R* = 0.437, *p* < .001, Figure 1b), showing a positive correlation. Subtotal cholecystectomy cases had significantly higher scores compared to complete cholecystectomy cases (20 [16–22] vs. 5 [2–11], *p* < .001, Figure 1c). Cases requiring open conversion had higher scores than those without conversion, although the difference was not statistically significant (14 [10–19] vs. 6 [2–13], *p* = .059, Figure 1d). Postoperatively, a significant positive correlation was observed between TGDS18 and length of hospital stay (*R* = 0.570, *p* < .001, Figure 1e). Scores were significantly higher in cases with complications greater than Clavien‐Dindo Grade 3a compared to those without (14 [12–16] vs. 6 [2–13], *p* = .048, Figure 1f). Cases complicated by postoperative intra‐abdominal abscess had higher scores compared to those without, but this was not statistically significant (17 [16, 17] vs. 6 [2–13], *p* = .071, Figure 1g).

### 3.3 Multivariate analysis for clinical findings associated with each sub‐score in TGDS18

Multivariate analysis of 69 cases with preoperative CT imaging ≤14 days identified clinical findings strongly correlated with each of the five TGDS18 sub‐scores: (a) gallbladder appearance, (b) Calot's triangle, (c) gallbladder bed, (d) additional gallbladder‐related findings, and (e) intra‐abdominal findings unrelated to inflammation (Table 2). For the sub‐score ‘around the gallbladder,’ significant clinical findings were a calcified stone in the cystic duct (*R* = 0.795, *p* = .013) and gallbladder drainage prior to LC (*R* = 1.070, *p* = .015). For the sub‐score ‘Calot's triangle,’ TG18 Grade ≥2 at onset (*R* = 1.903, *p* < .001) and a calcified stone in the cystic duct (*R* = 1.568, *p* < .001) were identified. For the sub‐score ‘gallbladder bed,’ TG18 Grade ≥2 at onset (*R* = 1.286, *p* < .001), a calcified stone in the cystic duct (*R* = 1.086, *p* < .001), and gallbladder drainage prior to LC (*R* = 1.343, *p* = .003) were significant. For the sub‐score ‘additional findings of the gallbladder and surroundings,’ a calcified stone in the cystic duct (*R* = 2.536, *p* = .002), urgent operation (*R* = 2.905, *p* = .006), and pericholecystic inflammation (*R* = 1.719, *p* = .044) were associated. CT findings of a calcified stone in the cystic duct and pericholecystic inflammation were obtained from plain CT scans performed within 14 days before surgery. For the sub‐score ‘intra‐abdominal findings unrelated to inflammation,’ aa‐CCI was significant (*R* = 0.111, *p* = .042).

**TABLE 2:** Multivariate analysis for clinical findings associated with each difficulty sub‐score.

	Correlation coefficient	95% CI	*p*‐Value
Score: Around the gallbladder			
CT finding: A calcified stone in the cystic duct^a^	0.795	0.177–1.414	.013
Gallbladder drainage prior to LC	1.070	0.217–1.923	.015
Score: Calot's triangle			
TG18 Grade ≥2 at onset	1.903	1.204–2.603	<.001
CT finding: A calcified stone in the cystic duct^a^	1.568	0.846–2.290	<.001
Score: Gallbladder bed			
TG18 Grade ≥2 at onset	1.286	0.589–1.982	<.001
CT finding: A calcified stone in the cystic duct^a^	1.086	0.459–1.714	<.001
Gallbladder drainage prior to LC	1.343	0.480–2.206	.003
Score: Additional findings of the gallbladder and its surroundings			
CT finding: A calcified stone in the cystic duct^a^	2.536	0.993–4.079	.002
Urgent operation	2.905	0.843–4.966	.006
CT finding: Pericholecystic inflammation^a^	1.719	0.051–3.386	.044
Score: Unrelated to inflammation			
Age‐adjusted Charlson comorbidity index	0.111	0.004–0.218	.042

Abbreviation: TG18, Tokyo Guidelines 2018.

^a^ CT findings were obtained from plain CT scans performed within 14 days before surgery.

### 3.4 Clinical factors increasing each difficulty sub‐score

**FIGURE 2:**
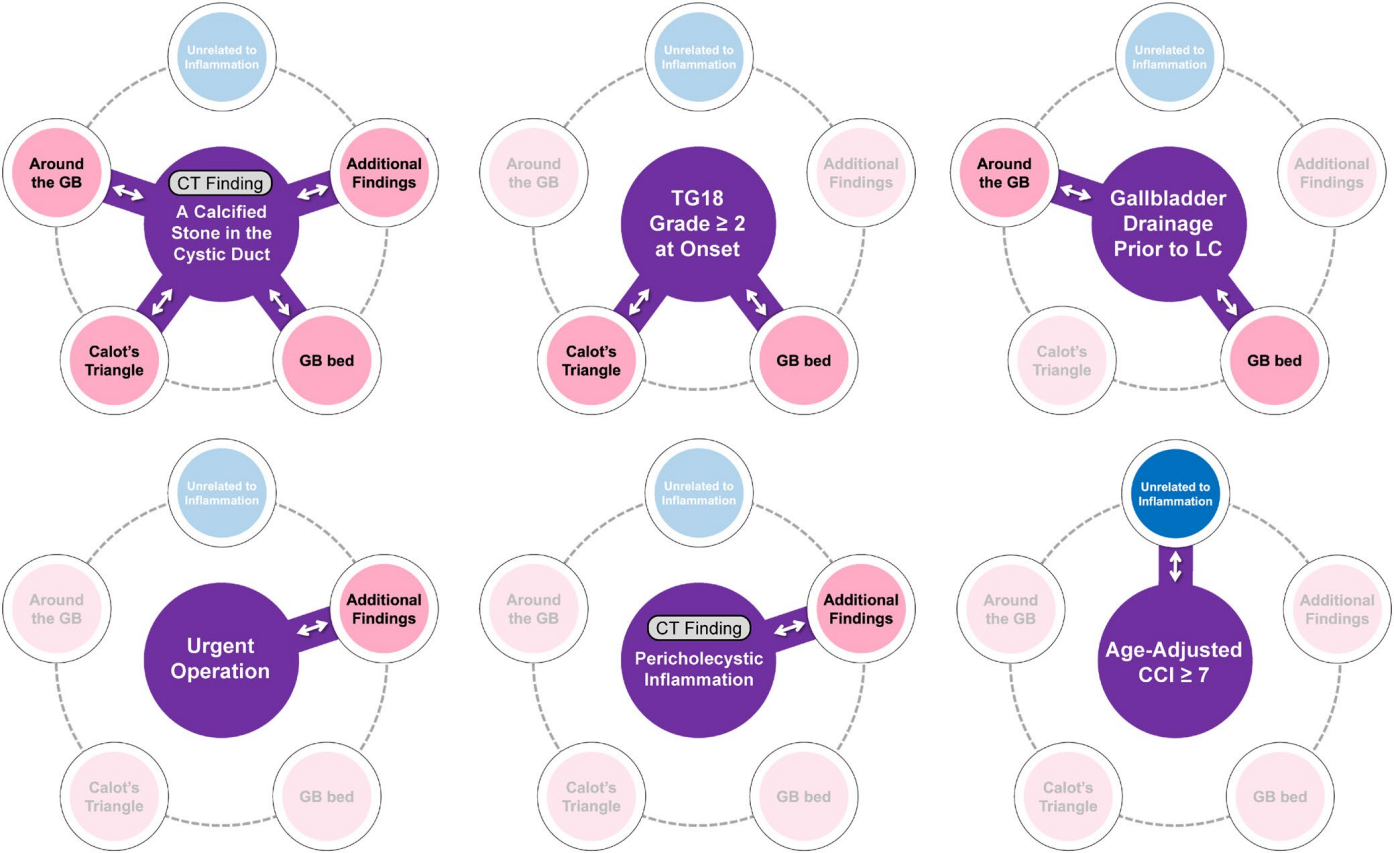
Association between six clinical preoperative findings and the difficulty sub‐scores. TGDS18 consists of the following sub‐scores: Appearance around the gallbladder, appearance of Calot's triangle, appearance of the gallbladder bed, additional findings related to the gallbladder and surroundings, and intra‐abdominal findings unrelated to inflammation. The analysis identified six preoperative clinical findings strongly associated with TGDS18 sub‐scores: A calcified stone in the cystic duct, TG18 Grade 2 or higher at onset, gallbladder drainage prior to LC, urgent operation, pericholecystic inflammation, and a higher age‐adjusted CCI (≥7). The relationships between these findings and TGDS18 sub‐scores were illustrated as key connections. CT findings of a calcified stone in the cystic duct and pericholecystic inflammation were obtained from plain CT scans performed within 14 days before surgery. CCI, Charlson comorbidity index; LC, laparoscopic cholecystectomy; TGDS18, surgical difficulty score from Tokyo Guidelines 2018; TG18, Tokyo Guidelines 2018.

**FIGURE 3:**
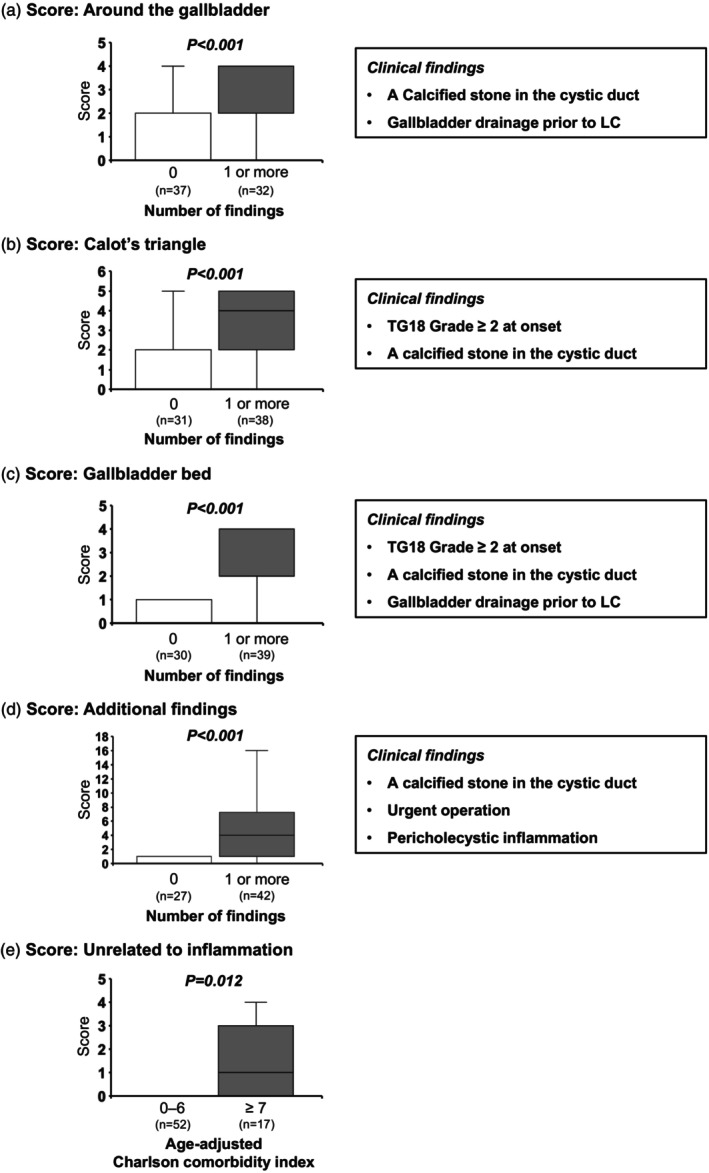
Clinical factors increasing each difficulty sub‐score. We compared the difficulty scores between patients with and without at least one clinical factor associated with each TGDS18 sub‐score. Patients who had these factors exhibited significantly higher scores across all sub‐score categories, compared to those without factors: (a) For the sub‐score ‘around the gallbladder,’ median score of 2 vs. 0 (*p* < .001); (b) for ‘Calot's triangle,’ 4 vs. 0 (*p* < .001); (c) for ‘gallbladder bed,’ 4 vs. 0 (*p* < .001); (d) for ‘additional findings of the gallbladder and its surroundings,’ 4 vs. 0 (*p* < .001); and (e) for ‘intra‐abdominal findings unrelated to inflammation,’ 1 vs. 0 (*p* = .012). CT findings of a calcified stone in the cystic duct and pericholecystic inflammation were obtained from plain CT scans performed within 14 days before surgery. TGDS18, surgical difficulty score from Tokyo Guidelines 2018; TG18, Tokyo Guidelines 2018.

Six preoperative clinical findings strongly associated with TGDS18 sub‐scores were identified, as shown in Figure 2. A calcified stone in the cystic duct, TG18 Grade 2 or higher at onset, and gallbladder drainage prior to LC were linked to multiple elevated sub‐score categories. Difficulty scores were compared between patients with and without at least one of these clinical findings across all sub‐score categories (Figure 3). For the sub‐score “around the gallbladder,” the scores were 2 [2–4] in patients with factors, compared to 0 [0–2] in those without (*p* < .001). For the sub‐score “Calot's triangle,” the scores were 4 [2–5] in patients with factors, compared to 0 [0–2] in those without (*p* < .001). For ‘gallbladder bed,’ the scores were 4 [2–4] in patients with factors, compared to 0 [0–1] in those without (*p* < .001). For ‘additional findings of the gallbladder and its surroundings,’ the scores were 4 [1–7] in patients with factors, compared to 0 [0–1] in those without (*p* < .001). For ‘intra‐abdominal findings unrelated to inflammation,’ the scores were 1 [0–3] in patients with aa‐CCI ≥7, compared to 0 [0–1] in those with aa‐CCI <6 (*p* = .012). In the analyzed cohort, aa‐CCI had a median of 4 [3–6], with a minimum of 0 and a maximum of 11. To identify patients with high aa‐CCI, optimal cut‐off values were explored using thresholds above the median (≤4 vs. ≥5, ≤5 vs. ≥6, and so on up to ≤10 vs. ≥11). We adopted a cut‐off value of 7, as it yielded the highest statistical significance using the Mann–Whitney *U* test (*Z* score = −2.517, *p* = .012).

### 3.5 Prediction of subtotal cholecystectomy requirement based on clinical findings related to the “Calot's triangle” sub‐score

**FIGURE 4:**
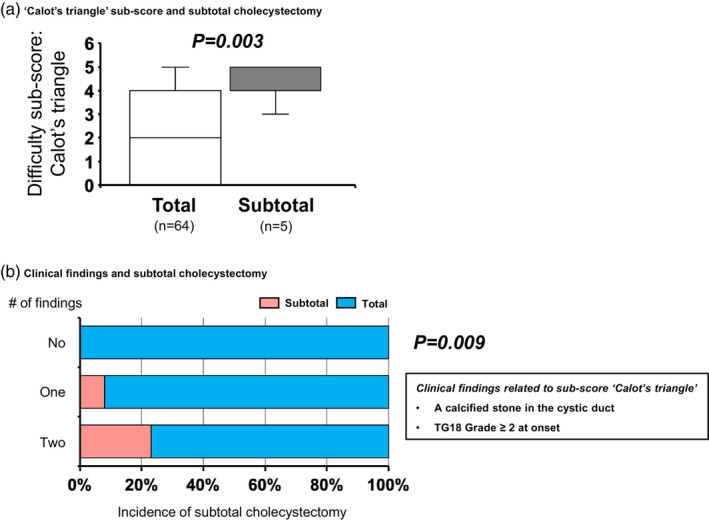
Prediction of subtotal cholecystectomy requirement based on clinical findings related to the ‘Calot's triangle’ sub‐score. (a) The difficulty sub‐score for ‘Calot's triangle’ was significantly higher in cases requiring subtotal cholecystectomy, compared to those undergoing total cholecystectomy (*p* = .003). (b) The rate of subtotal cholecystectomy increased proportionally with the number of two clinical factors associated with the elevated ‘Calot's triangle’ sub‐score: A calcified stone in the cystic duct and TG18 Grade 2 or higher at onset (0% in patients with no findings, 8% with one finding, and 23% with both findings, *p* = .009). TG18, Tokyo Guideliness 2018.

We evaluated the association between subtotal cholecystectomy and TGDS18 using a cohort of patients with CT imaging. The difficulty sub‐score for “Calot's triangle” in cases requiring subtotal cholecystectomy had a median of 5 [5], significantly higher than the 2 [0–4] seen in total cholecystectomy cases (*p* = .003, Figure 4a). Among the clinical findings associated with TGDS18 (Figure 2), we focused on the findings linked to ‘Calot's triangle’ sub‐score: a calcified stone in the cystic duct and TG18 Grade 2 or higher at onset. The rate of subtotal cholecystectomy increased proportionally with the number of factors, with 23% of patients possessing both factors requiring subtotal cholecystectomy (0% in patients with no findings, 8% in patients with one finding, *p* = .009, Figure 4b).

### 3.6 Prediction of cholecystectomy with the posterior wall left behind on the gallbladder bed based on clinical findings related to the ‘gallbladder bed’ sub‐score

**FIGURE 5:**
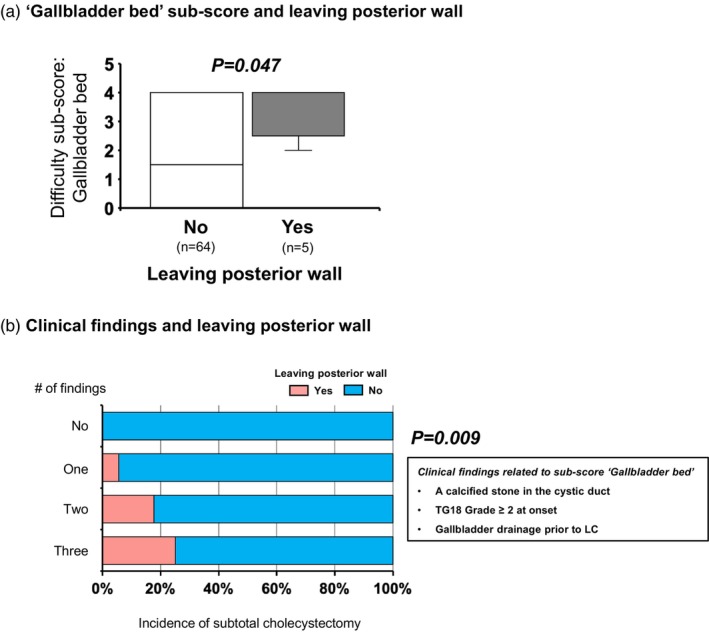
Prediction of cholecystectomy with the posterior wall left behind on the gallbladder bed based on clinical findings related to the “Gallbladder bed” sub‐score. (a) The difficulty sub‐score for the “Gallbladder bed” was significantly higher in cholecystectomy cases where the posterior wall was left behind, compared to those undergoing total cholecystectomy (*p* = .047). (b) The rate of cholecystectomy with the posterior wall left behind increased proportionally with the number of three clinical factors associated with the elevated “Gallbladder bed” sub‐score: A calcified stone in the cystic duct, TG18 Grade 2 or higher at onset, and gallbladder drainage prior to LC (0% for no factors, 6% for one factor, 18% for two factors, and 25% for three factors, *p* = .009). LC, laparoscopic cholecystectomy; TG18, Tokyo Guidelines 2018.

To avoid liver injury, some challenging cases may require leaving part of the gallbladder's posterior wall intact. We evaluated the association between this approach and TGDS18 in patients with CT imaging. Cases where the posterior wall was left behind had a significantly higher median difficulty sub‐score for the ‘Gallbladder bed’ (4 [3, 4]) compared to cases where it was not left (2 [0–4], *p* = .047, Figure 5a). The rate of cholecystectomy with posterior wall left behind increased proportionally with the multiple clinical findings related to ‘Gallbladder bed’ sub‐score: a calcified stone in the cystic duct, TG18 Grade 2 or higher at onset, and gallbladder drainage prior to LC, reaching 25% in patients with all findings (0% for no factors, 6% for one factor, 18% for two factors, *p* = .009, Figure 5b).

## 4 DISCUSSION

This study is the first, to our knowledge, to comprehensively evaluate the relationship between preoperative clinical findings and the Surgical Difficulty Score in LC. Notably, we identified six clinical findings strongly associated with each sub‐score of the TGDS18. By referring to these findings, we were preoperatively able to predict high‐difficulty cases including those requiring subtotal cholecystectomy.

Acute cholecystitis presents with a wide spectrum of clinical presentations, and the surgical difficulty varies accordingly. TGDS18, with its objective scoring system, eliminates subjective bias among surgeons and allows for comparison of difficulty across cases. This significantly enhances our understanding of which cases may be more challenging. Predicting the surgical difficulty is crucial in determining the treatment strategy for acute cholecystitis, especially since LC is often a gateway for junior surgeons. Therefore, matching cases to the skill level of the surgeon is highly valuable. The more challenging the surgery, the worse the surgical outcomes tend to be; and thus, we believe that proper evaluation of surgical difficulty can better manage both intra‐ and postoperative outcomes. Higher TGDS18 scores have been shown to correlate with longer operative times, greater blood loss, and higher rates of subtotal cholecystectomy, as demonstrated in this study and in prior research by Asai et al. and Egawa et al.^15^, ^22^ We further showed that higher TGDS18 scores were linked to longer postoperative stays and more complications (Clavien‐Dindo Grade 3A or higher), confirming TGDS18 as a reliable indicator of surgical difficulty.

Achieving the CVS^23^ is the most critical step to safely completing LC while avoiding BDI. Since the difficulty of attaining CVS depends on the condition of local structures (gallbladder surroundings, Calot's triangle, gallbladder bed), TGDS18, which allows localized assessment of surgical difficulty, is a highly practical tool. However, its limitation lies in the lack of preoperative calculability. In contrast, laparoscopic liver resection employs scoring systems based solely on preoperative factors, enabling better preoperative evaluation and preparation.^24^ To address this limitation in LC, we aimed to identify preoperative clinical findings correlated with TGDS18 sub‐scores. Multivariate analysis identified the six preoperative factors correlated with each TGDS18 sub‐score: a calcified cystic duct stone, gallbladder drainage, TG18 Grade 2 or higher at onset, emergency surgery, pericholecystic inflammation, and aa‐CCI ≥7 (Table 2). As shown in Figure 2, the presence of cystic duct stones increased all sub‐scores associated with inflammation, which is unsurprising given that cholecystitis is caused by obstruction of the cystic duct. Literature also reports that gallbladder obstruction due to gallstones worsens surgical outcomes.^25^ Preoperative gallbladder drainage was associated with higher sub‐scores in the gallbladder bed and surroundings of the gallbladder, likely because all drainage procedures in this cohort were performed via the gallbladder bed, leading to fibrosis, scarring, and adhesions near the fistula site. Sub‐scores correlated with TG18 Grade were Calot's triangle and the gallbladder bed. As the Grade progresses, the surgical difficulty increased and the risk of bile duct injury rose, which was previously reported.^26^ The “Additional findings” sub‐score encompasses general acute inflammatory findings of the gallbladder and its surroundings caused by cholecystitis, without being limited to specific areas such as Calot's triangle or the gallbladder bed. The clinical findings correlated with this sub‐score were urgent operation and pericholecystic inflammation, both of which clearly indicate the presence of acute inflammatory findings at the time of surgery. The sub‐score “unrelated to inflammation” was correlated with the age‐adjusted CCI. Given the higher scores for cirrhosis‐related items in this sub‐score and the aging and various comorbidities in cirrhotic patients,^27^, ^28^ a high aa‐CCI may indicate cirrhosis (Figure S5). Our institution is a leading center for liver disease treatment and performs more LC on patients with cirrhosis than the average, so further validation is needed before generalizing this result.

As a clinical application of findings associated with TGDS18, we propose a method to predict the need for subtotal cholecystectomy preoperatively. Subtotal cholecystectomy is a standardized bailout procedure for difficult cholecystitis cases to prevent BDI. However, establishing criteria for its indication has remained challenging despite several previous studies.^29^, ^30^ As also noted by Asai et al.,^16^ because a higher Calot's triangle sub‐score is a risk factor for subtotal cholecystectomy (Figure 4a), we hypothesized that preoperative clinical findings associated with Calot's triangle difficulty—such as a calcified stone in the cystic duct and TG18 Grade 2 or higher—could predict the risk of requiring subtotal cholecystectomy, a hypothesis that was confirmed. Notably, the presence of multiple clinical findings cumulatively increased this risk (Figure 4b). This intuitive and straightforward approach demonstrates greater applicability than conventional risk assessments, with potential extensions to other surgical risk predictions. For example, in cases where part of the posterior gallbladder wall is left on the gallbladder bed to avoid liver injury, a higher gallbladder bed sub‐score is anticipated. Accordingly, we were able to predict this risk by referencing the preoperative clinical findings that correlate with the gallbladder bed sub‐score (Figure 5).

This study has several limitations, including its single‐center, retrospective design and small sample size, further impacted by the limited surgical video data. This was due to our facility's storage system (older video files overwritten due to HDD capacity), resulting in a maximum of 106 analyzable cases. Additionally, in the analysis of the association between clinical findings and TGDS18, 37 cases without preoperative CT were excluded. In all LC cases at our institution, biliary anatomy and stones are preoperatively evaluated with magnetic resonance or direct cholangiography. Of 46 cases with no stones on preoperative CT, 5 had radiolucent stones, but cholangiography was not always performed close to surgery. This study focused on calcified (radiopaque) stones, assessable by CT, and did not evaluate radiolucent stones. The TGDS18 in this study were reviewed by a single surgeon, with good inter‐rater agreement confirmed statistically with another surgeon's assessment. Previous studies have also shown TGDS18's high objectivity across evaluators.^22^ The primary goal of this study was to identify clinical findings associated with TGDS18 and to propose a novel framework, the value of which transcends the stated limitations. Future multi‐center studies are warranted to validate its broader applicability.

In conclusion, this study was the first to identify preoperative clinical factors associated with the TG18 Surgical Difficulty Score. Referencing clinical findings linked to TGDS18 sub‐scores enables preoperative prediction of high‐difficulty cases and the development of tailored surgical strategies, providing a simple tool to improve risk management in LC for acute cholecystitis.

## CONFLICT OF INTEREST STATEMENT

All authors declare no conflict of interest for this article.

## Supporting information


Data S1

## References

[1] Diamond IR , Grant RC , Feldman BM , Pencharz PB , Ling SC , Moore AM , et al. Defining consensus: a systematic review recommends methodologic criteria for reporting of Delphi studies. J Clin Epidemiol. 2014;67(4):401–409. 10.1016/j.jclinepi.2013.12.002 24581294

[2] Schrenk P , Woisetschläger R , Rieger R , Wayand WU . A diagnostic score to predict the difficulty of a laparoscopic cholecystectomy from preoperative variables. Surg Endosc. 1998;12:148–150. 9479730 10.1007/s004649900616

[3] Sakuramoto S , Sato S , Okuri T , Sato K , Hiki Y , Kakita A . Preoperative evaluation to predict technical difficulties of laparoscopic cholecystectomy on the basis of histological inflammation findings on resected gallbladder. Am J Surg. 2000;179:114–121. 10773146 10.1016/s0002-9610(00)00248-8

[4] Hiromatsu T , Hasegawa H , Sakamoto E , Komatsu S , Kawai K , Tabata S , et al. Preoperative evaluation of difficulty on laparoscopic cholecystectomy. Jpn J Gastroenterol Surg. 2007;40:1449–1455.

[5] Lal P , Agarwal PN , Malik VK , Chakravarti AL . A difficult laparoscopic cholecystectomy that requires conversion to open procedure can be predicted by preoperative ultrasonography. JSLS. 2002;6:59–63. 12002299 PMC3043388

[6] Kama NA , Kologlu M , Doganay M , Reis E , Atli M , Dolapci M . A risk score for conversion from laparoscopic to open cholecystectomy. Am J Surg. 2001;181:520–525. 11513777 10.1016/s0002-9610(01)00633-x

[7] Lipman JM , Claridge JA , Haridas M , Martin MD , Yao DC , Grimes KL , et al. Preoperative findings predict conversion from laparoscopic to open cholecystectomy. Surgery. 2007;142(4):556–565. 10.1016/j.surg.2007.07.010 17950348

[8] Rosen M , Brody F , Ponsky J . Predictive factors for conversion of laparoscopic cholecystectomy. Am J Surg. 2002;184:254–258. 12354595 10.1016/s0002-9610(02)00934-0

[9] Simopoulos C , Botaitis S , Polychronidis A , Tripsianis G , Karayiannakis AJ . Risk factors for conversion of laparoscopic cholecystectomy to open cholecystectomy. Surg Endosc Other Interv Tech. 2005;19:905–909. 10.1007/s00464-004-2197-0 15868267

[10] Nassar AHM , Ashkar KA , Mohamed AY , Hafiz AA . Is laparoscopic cholecystectomy possible without video technology? Minim Invasive Ther. 1995;4(2):63–65. 10.3109/13645709509152757

[11] Griffiths EA , Hodson J , Vohra RS , Marriott P , the CholeS Study Group , Katbeh T , et al. Utilisation of an operative difficulty grading scale for laparoscopic cholecystectomy. Surg Endosc. 2019;33:110–121. 29956029 10.1007/s00464-018-6281-2 PMC6336748

[12] Nassar AHM , Hodson J , Ng HJ , Vohra RS , Katbeh T , Zino S , et al. Predicting the difficult laparoscopic cholecystectomy: development and validation of a pre‐operative risk score using an objective operative difficulty grading system. Surg Endosc. 2020;34:4549–4561. 31732855 10.1007/s00464-019-07244-5

[13] Ng HJ , Ahmed Z , Khan KS , Katbeh T , Nassar AHM . C‐reactive protein level as a predictor of difficult emergency laparoscopic cholecystectomy. BJS Open. 2019;3:641–645. 31592082 10.1002/bjs5.50189 PMC6773624

[14] Nassar AHM , Zanati HE , Ng HJ , Khan KS , Wood C . Open conversion in laparoscopic cholecystectomy and bile duct exploration: subspecialisation safely reduces the conversion rates. Surg Endosc. 2022;36:550–558. 33528666 10.1007/s00464-021-08316-1 PMC8741693

[15] Egawa N , Miyoshi A , Manabe T , Sadashima E , Koga H , Sato H , et al. Clinical evaluation of a surgical difficulty score for laparoscopic cholecystectomy for acute cholecystitis proposed in the Tokyo Guidelines 2018. J Hepatobiliary Pancreat Sci. 2023;30:625–632. 36287104 10.1002/jhbp.1258

[16] Asai K , Iwashita Y , Ohyama T , Endo I , Hibi T , Umezawa A , et al. Application of a novel surgical difficulty grading system during laparoscopic cholecystectomy. J Hepatobiliary Pancreat Sci. 2022;29:758–767. 10.1002/jhbp.1068 34748289

[17] Okamoto K , Suzuki K , Takada T , Strasberg SM , Asbun HJ , Endo I , et al. Tokyo Guidelines 2018: flowchart for the management of acute cholecystitis. J Hepatobiliary Pancreat Sci. 2018;25(1):55–72. 10.1002/jhbp.516 29045062

[18] Charlson ME , Pompei P , Ales KL , MacKenzie CR . A new method of classifying prognostic comorbidity in longitudinal studies: development and validation. J Chronic Dis. 1987;40:373–383. 3558716 10.1016/0021-9681(87)90171-8

[19] Yokoe M , Hata J , Takada T , Strasberg SM , Asbun HJ , Wakabayashi G , et al. Tokyo Guidelines 2018: diagnostic criteria and severity grading of acute cholecystitis (with videos). J Hepatobiliary Pancreat Sci. 2018;25:41–54. 29032636 10.1002/jhbp.515

[20] Wakabayashi G , Iwashita Y , Hibi T , Takada T , Strasberg SM , Asbun HJ , et al. Tokyo Guidelines 2018: surgical management of acute cholecystitis: safe steps in laparoscopic cholecystectomy for acute cholecystitis (with videos). J Hepatobiliary Pancreat Sci. 2018;25(1):73–86. 10.1002/jhbp.517 29095575

[21] Koo TK , Li MY . A guideline of selecting and reporting intraclass correlation coefficients for reliability research. J Chiropr Med. 2016;15:155–163. 27330520 10.1016/j.jcm.2016.02.012 PMC4913118

[22] Asai K , Ohyama T , Watanabe M , Moriyama H , Kujiraoka M , Watanabe R , et al. Validation of a surgical difficulty grading system in laparoscopic cholecystectomy for acute cholecystitis. J Hepatobiliary Pancreat Sci. 2024;31(2):80–88. 10.1002/jhbp.1367 37803518

[23] Strasberg SM , Brunt LM . Rationale and use of the critical view of safety in laparoscopic cholecystectomy. J Am Coll Surg. 2010;211:132–138. 20610259 10.1016/j.jamcollsurg.2010.02.053

[24] Ban D , Tanabe M , Ito H , Otsuka Y , Nitta H , Abe Y , et al. A novel difficulty scoring system for laparoscopic liver resection. J Hepatobiliary Pancreat Sci. 2014;21:745–753. 25242563 10.1002/jhbp.166

[25] Gupta N , Ranjan G , Arora MP , Goswami B , Chaudhary P , Kapur A , et al. Validation of a scoring system to predict difficult laparoscopic cholecystectomy. Int J Surg. 2013;11:1002–1006. 23751733 10.1016/j.ijsu.2013.05.037

[26] Törnqvist B , Waage A , Zheng Z , Ye W , Nilsson M . Severity of acute cholecystitis and risk of iatrogenic bile duct injury during cholecystectomy, a population‐based case–control study. World J Surg. 2016;40(5):1060–1067. 10.1007/s00268-015-3365-1 26669783

[27] Chan W‐K , Chuah K‐H , Rajaram RB , Lim LL , Ratnasingam J , Vethakkan SR . Metabolic dysfunction‐associated steatotic liver disease (MASLD): a state‐of‐the‐art review. J Obes Metab Syndr. 2023;32:197–213. 37700494 10.7570/jomes23052 PMC10583766

[28] Kaur H , Premkumar M . Diagnosis and management of cirrhotic cardiomyopathy. J Clin Exp Hepatol. 2022;12:186–199. 35068798 10.1016/j.jceh.2021.08.016 PMC8766707

[29] da Costa DW , Schepers NJ , Bouwense SA , Hollemans RA , van Santvoort HC , Bollen TL , et al. Predicting a ‘difficult cholecystectomy’ after mild gallstone pancreatitis. HPB. 2019;21:827–833. 30538063 10.1016/j.hpb.2018.10.015

[30] Tomihara H , Tomimaru Y , Hashimoto K , Fukuchi N , Yokoyama S , Mori T , et al. Preoperative risk score to predict subtotal cholecystectomy after gallbladder drainage for acute cholecystitis: secondary analysis of data from a multi‐institutional retrospective study (CSGO‐HBP‐017B). Asian J Endosc Surg. 2022;15:555–562. 35302288 10.1111/ases.13051

