# Supplementary material for: A new framework for tailoring laparoscopic cholecystectomy: Integrating preoperative clinical factors with surgical difficulty based on the Tokyo Guidelines 2018
Source: J Hepatobiliary Pancreat Sci. 2025 Apr 10;32(6):452–64. doi: 10.1002/jhbp.12145 (PMC12188173; doi:10.1002/jhbp.12145)
Supplement: Supplementary file 1 — Data S1 [file JHBP-32-452-s001.docx]

**Supplemental Materials**

**Title:**

**A New Framework for Tailoring Laparoscopic Cholecystectomy: Integrating Preoperative Clinical Factors with Surgical Difficulty Based on the Tokyo Guidelines 2018**

**Authors:**

Daisuke Noguchi^1^, Aoi Hayasaki^1^, Takahiro Ito^1^, Yusuke Iizawa^1^, Takehiro Fujii^1^, Akihiro Tanemura^1^, Yasuhiro Murata^1^, ^,^ Naohisa Kuriyama^1^,Masashi Kishiwada^1^, Shugo Mizuno^1^

**Author affiliations:**

1. Department of Hepatobiliary Pancreatic and Transplant Surgery, Mie University Graduate School of Medicine

**Contents:** (Page) – Figure / Table

**(2) – Table S1.** Inter-rater agreement of surgical difficulty assessment between evaluators: The intraclass correlation coefficient

**(3) – Table S2.** Reviewed preoperative variables in analysis of association with difficulty score

**(4) – Table S3.** Univariate analysis for clinical findings associated with each difficulty sub-score: Continuous variables

**(5) – Table S4.** Univariate analysis for clinical findings associated with each difficulty sub-score: Categorized variables

**(7) – Figure S1.** Study flow

**(8) – Figure S2.** Preoperative CT findings

**(9) – Figure S3.** The surgical difficulty score from the Tokyo guideline 2018 (TGDS18)

**(10) – Figure S4.** Inter-rater agreement of surgical difficulty assessment between evaluators: The Bland-Altman plot for the total score

**(11) – Figure S5.** Charlson comorbidity index and liver cirrhosis

**Table S1. Inter-rater agreement of surgical difficulty assessment between evaluators: The intraclass correlation coefficient**

| **Score** | **ICC*** | **95%CI** | | | ***p-value*** |
| --- | --- | --- | --- | --- | --- |
| **Around the gallbladder** | 0.865 | 0.782 | – | 0.917 | ***<0.001*** |
| **Calot’s triangle** | 0.912 | 0.858 | – | 0.945 | ***<0.001*** |
| **Gallbladder bed** | 0.925 | 0.878 | – | 0.953 | ***<0.001*** |
| **Additional findings of the gallbladder**  **and its surroundings** | 0.883 | 0.810 | – | 0.927 | ***<0.001*** |
| **Unrelated to inflammatory findings** | 0.898 | 0.835 | – | 0.937 | ***<0.001*** |
| **Total score (TGDS18)** | 0.963 | 0.940 | – | 0.977 | ***<0.001*** |

* ICC values less than 0.5, between 0.5 and 0.75, between 0.75 and 0.9, and greater than 0.90 indicate poor, moderate, good, and excellent agreement, respectively.

Abbreviations: CI, confidence Interval; ICC, intraclass correlation coefficient; TGDS18, surgical difficulty score from Tokyo guideline 2018

**Table S2. Reviewed preoperative variables in analysis of association with difficulty score**

| **Reviewed variables in cohort with CT image within 14 days (n=69)** |
| --- |
| Age (y.o.) |
| BMI (kg/m2) |
| Age-adjusted Charlson comorbidity index |
| Previous abdominal operation (%) |
| Interval between onset and LC (day) |
| Urgent operation (%) |
| Symptoms at onset (%) |
| High fever ≥ 38 degree |
| Murphy’s sign |
| Cholecystitis severity at onset |
| TG18 Grade ≥ 2 |
| Pericholecystic or liver abscess |
| Blood test at onset |
| WBC (/µL) |
| CRP (mg/dL) |
| Intervention before operation (%) |
| Gallbladder drainage |
| Endoscopic lithotomy |
| Preoperative CT findings within 14 days (%) |
| A calcified stone in the cystic duct |
| Pericholecystic fluid |
| Pericholecystic inflammation |
| Recent preoperative blood test |
| WBC (/µL) |
| CRP (mg/dL) |

Abbreviations: BMI, body mass index; LC, laparoscopic cholecystectomy; TG18, Tokyo guideline 2018; WBC, white blood cell; CRP, c-reactive protein

**Table S3. Univariate analysis for clinical findings associated with each difficulty sub-score: Continuous variables**

| **Variables** | **Around the gallbladder** | |  | **Calot’s triangle** | |  | **Gallbladder bed** | |  | **Additional findings**  **of the gallbladder**  **and its surroundings** | |  | **Unrelated to inflammation** | |
| --- | --- | --- | --- | --- | --- | --- | --- | --- | --- | --- | --- | --- | --- | --- |
|  | **Correlation**  **Coefficient** | ***P-value*** |  | **Correlation**  **Coefficient** | ***P-value*** |  | **Correlation**  **Coefficient** | ***P-value*** |  | **Correlation**  **Coefficient** | ***P-value*** |  | **Correlation**  **Coefficient** | ***P-value*** |
| **Age (y.o.)** | 0.353 | ***0.003*** |  | 0.326 | ***0.006*** |  | 0.294 | ***0.014*** |  | 0.127 | *0.297* |  | 0.076 | *0.537* |
| **BMI (kg/m2)** | -0.148 | *0.223* |  | -0.185 | *0.128* |  | -0.071 | *0.560* |  | -0.100 | *0.415* |  | -0.023 | *0.854* |
| **Age-adjusted Charlson comorbidity index** | 0.324 | ***0.007*** |  | 0.222 | *0.066* |  | 0.194 | *0.110* |  | 0.171 | *0.160* |  | 0.248 | ***0.040*** |
| **Interval between onset and LC (day)** | -0.090 | *0.463* |  | -0.193 | *0.112* |  | -0.005 | *0.969* |  | -0.380 | ***0.001*** |  | -0.109 | *0.374* |
| **Blood test at onset** |  |  |  |  |  |  |  |  |  |  |  |  |  |  |
| **WBC (/µL)** | 0.293 | ***0.016*** |  | 0.269 | ***0.028*** |  | 0.377 | ***0.002*** |  | 0.247 | ***0.044*** |  | -0.096 | *0.440* |
| **CRP (mg/dL)** | 0.488 | ***<0.001*** |  | 0.526 | ***<0.001*** |  | 0.562 | ***<0.001*** |  | 0.374 | ***0.002*** |  | -0.092 | *0.463* |
| **Recent preoperative blood test** |  |  |  |  |  |  |  |  |  |  |  |  |  |  |
| **WBC (/µL)** | 0.012 | *0.921* |  | -0.065 | *0.595* |  | -0.090 | *0.462* |  | 0.211 | *0.082* |  | 0.105 | *0.389* |
| **CRP (mg/dL)** | 0.351 | ***0.003*** |  | 0.341 | ***0.004*** |  | 0.220 | *0.069* |  | 0.514 | ***<0.001*** |  | 0.092 | *0.451* |

Abbreviations: BMI, body mass index; CRP, c-reactive protein; LC, laparoscopic cholecystectomy; WBC, white blood cell

**Table S4. Univariate analysis for clinical findings associated with each difficulty sub-score: Categorized variables**

| **Variables** | **Around the gallbladder** | |  | **Calot’s triangle** | |  | **Gallbladder bed** | |  | **Additional findings**  **of the gallbladder**  **and its surroundings** | |  | **Unrelated to inflammation** | |
| --- | --- | --- | --- | --- | --- | --- | --- | --- | --- | --- | --- | --- | --- | --- |
|  | **Score [IQR]** | ***P-value*** |  | **Score [IQR]** | ***P-value*** |  | **Score [IQR]** | ***P-value*** |  | **Score [IQR]** | ***P-value*** |  | **Score [IQR]** | ***P-value*** |
| **Previous abdominal operation** |  | *0.394* |  |  | *0.217* |  |  | *0.176* |  |  | *0.785* |  |  | *0.988* |
| No | 2 [0–2] |  |  | 2 [0–4] |  |  | 3 [0–4] |  |  | 1 [0–4] |  |  | 0 [0–1] |  |
| Yes | 2 [0–2] |  |  | 2 [0–4] |  |  | 2 [0–3] |  |  | 1 [0–4] |  |  | 0 [0–1] |  |
| **Urgent operation** |  | *0.311* |  |  | ***0.018*** |  |  | *0.831* |  |  | ***<0.001*** |  |  | *0.190* |
| No | 2 [0–2] |  |  | 2 [0–4] |  |  | 2 [0–4] |  |  | 1 [0–4] |  |  | 0 [0–1] |  |
| Yes | 2 [0–2] |  |  | 3 [2–4] |  |  | 2 [1–3] |  |  | 7 [4–8] |  |  | 0 [0–2] |  |
| **Symptoms at onset** |  |  |  |  |  |  |  |  |  |  |  |  |  |  |
| **High fever ≥ 38 degree** |  | ***0.004*** |  |  | ***<0.001*** |  |  | ***<0.001*** |  |  | ***0.003*** |  |  | *0.309* |
| No | 0 [0–2] |  |  | 2 [0–3] |  |  | 1 [0–3] |  |  | 1 [0–4] |  |  | 0 [0–2] |  |
| Yes | 2 [2–2] |  |  | 4 [3–4] |  |  | 4 [3–4] |  |  | 4 [1–7] |  |  | 0 [0–0] |  |
| **Murphy’s sign** |  | ***0.030*** |  |  | ***0.001*** |  |  | ***<0.001*** |  |  | ***0.012*** |  |  | *0.466* |
| No | 0 [0–2] |  |  | 2 [0–3] |  |  | 1 [0–3] |  |  | 1 [0–4] |  |  | 0 [0–1] |  |
| Yes | 2 [2–2] |  |  | 4 [2–5] |  |  | 4 [2–4] |  |  | 4 [1–4] |  |  | 0 [0–1] |  |
| **Cholecystitis severity at onset** |  |  |  |  |  |  |  |  |  |  |  |  |  |  |
| **TG18 Grade ≥ 2** |  | ***<0.001*** |  |  | ***<0.001*** |  |  | ***<0.001*** |  |  | ***<0.001*** |  |  | ***<0.001*** |
| No | 0 [0–2] |  |  | 0 [0–2] |  |  | 1 [0–2] |  |  | 0 [0–1] |  |  | 0 [0–1] |  |
| Yes | 2 [2–4] |  |  | 4 [3–5] |  |  | 4 [3–4] |  |  | 4 [2–8] |  |  | 0 [0–1] |  |
| **Pericholecystic or liver abscess** |  | ***0.045*** |  |  | ***<0.001*** |  |  | ***<0.001*** |  |  | ***0.033*** |  |  | *0.591* |
| No | 2 [0–2] |  |  | 2 [0–3] |  |  | 1 [0–3] |  |  | 1 [0–4] |  |  | 0 [0–1] |  |
| Yes | 2 [2–2] |  |  | 4 [4–5] |  |  | 4 [4–4] |  |  | 4 [1–7] |  |  | 0 [0–0] |  |
| **Intervention before operation** |  |  |  |  |  |  |  |  |  |  |  |  |  |  |
| **Gallbladder drainage** |  | ***<0.001*** |  |  | ***0.005*** |  |  | ***<0.001*** |  |  | *0.481* |  |  | *0.206* |
| No | 1 [0–2] |  |  | 2 [0–3] |  |  | 1 [0–3] |  |  | 1 [0–4] |  |  | 0 [0–2] |  |
| Yes | 2 [2–4] |  |  | 4 [2–5] |  |  | 4 [3–4] |  |  | 3 [1–4] |  |  | 0 [0–0] |  |
| **Endoscopic lithotomy** |  | *0.608* |  |  | *0.455* |  |  | *0.830* |  |  | *0.174* |  |  | *0.186* |
| No | 2 [0–2] |  |  | 3 [0–4] |  |  | 2 [0–4] |  |  | 2 [0–4] |  |  | 0 [0–2] |  |
| Yes | 2 [0–2] |  |  | 2 [0–3] |  |  | 3 [1–4] |  |  | 1 [0–1] |  |  | 0 [0–0] |  |
| **Preoperative CT findings within 14 days** |  |  |  |  |  |  |  |  |  |  |  |  |  |  |
| **A calcified stone in the cystic duct** |  | ***0.005*** |  |  | ***<0.001*** |  |  | ***<0.001*** |  |  | ***<0.001*** |  |  | *0.189* |
| No | 1 [0–2] |  |  | 2 [0–3] |  |  | 1 [0–3] |  |  | 1 [0–3] |  |  | 0 [0–1] |  |
| Yes | 2 [2–4] |  |  | 4 [3–5] |  |  | 4 [2–4] |  |  | 4 [1–8] |  |  | 0 [0–2] |  |
| **Pericholecystic fluid** |  | ***0.031*** |  |  | *0.095* |  |  | *0.185* |  |  | ***0.009*** |  |  | *0.987* |
| No | 2 [0–2] |  |  | 2 [0–4] |  |  | 2 [0–4] |  |  | 1 [0–4] |  |  | 0 [0–1] |  |
| Yes | 3 [2–4] |  |  | 4 [3–5] |  |  | 3 [2–4] |  |  | 7 [5–10] |  |  | 0 [0–2] |  |
| **Pericholecystic inflammation** |  | ***0.005*** |  |  | ***<0.001*** |  |  | ***0.039*** |  |  | ***<0.001*** |  |  | *0.391* |
| No | 0 [0–2] |  |  | 0 [0–3] |  |  | 1 [0–3] |  |  | 0 [0–1] |  |  | 0 [0–1] |  |
| Yes | 2 [1–3] |  |  | 3 [2–5] |  |  | 3 [1–4] |  |  | 4 [1–8] |  |  | 0 [0–1] |  |

Abbreviations: IQR, Interquartile Range; TG18, Tokyo guideline 2018

**Figure S1. Study flow**


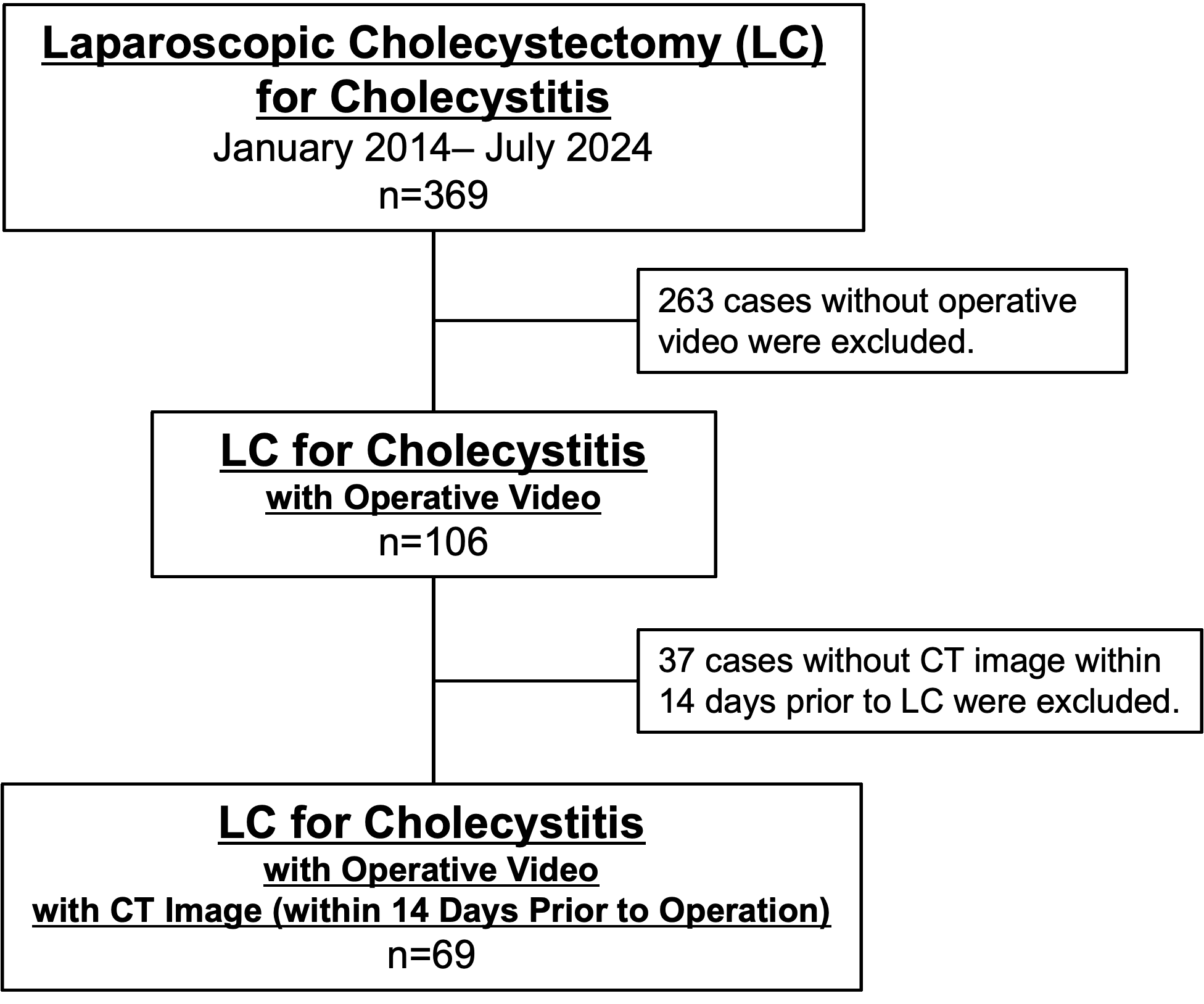


Abbreviations: CT, computed tomography; LC, laparoscopic cholecystectomy

**Figure S2. Preoperative CT findings**


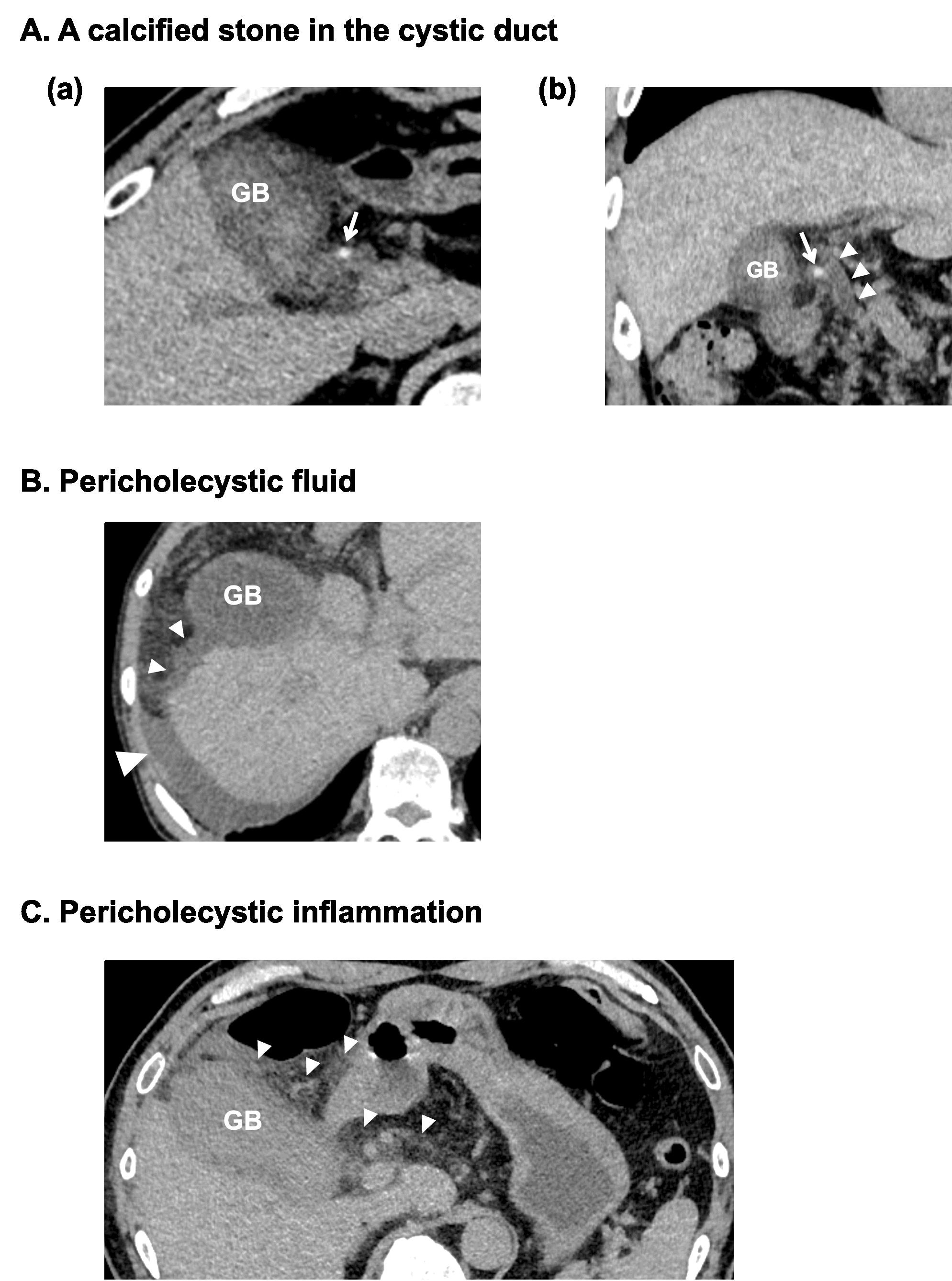


Preoperative imaging findings were based on plain CT scans performed within 14 days before surgery, which are widely used for diagnosing cholecystitis. In the analysis of the relationship between preoperative findings and TGDS18, we used the following CT findings: (A) A calcified (radiopaque) stone in the cystic duct. (a) In the axial plane, a stone (arrow) was observed in the cystic duct. (b) In the coronal plane, a stone (arrow) was located within the cystic duct, which connects the gallbladder (GB) to the common bile duct (arrowheads). (B) Pericholecystic fluid. Fluid (arrowheads) was observed around the GB, extending to the dorsal side of the liver. (C) Pericholecystic inflammation. Inflammation surrounding the GB was detected as pericholecystic fat stranding (arrowheads). Fat stranding indicates changes in adipose tissue properties, such as edema and fibrosis, as well as structural disruption, which appear as increased CT density and a reticulated pattern.

Abbreviations: CT, computed tomography; GB, gallbladder; TGDS18, surgical difficulty score from Tokyo guideline 2018

**Figure S3. The surgical difficulty score from the Tokyo guideline 2018 (TGDS18)**


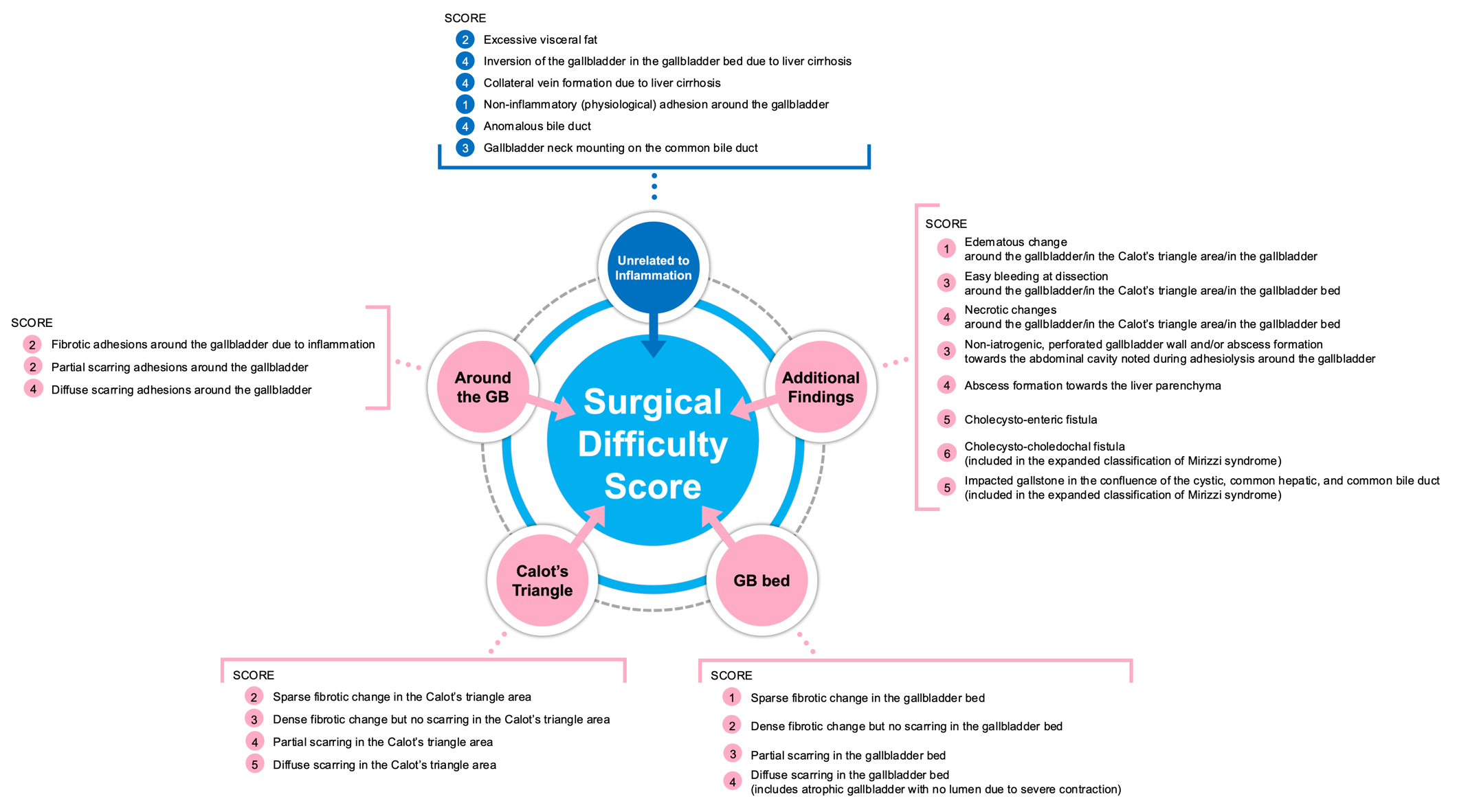


The Surgical Difficulty Score from the Tokyo Guidelines 2018 (TGDS18) evaluates intraoperative findings across 25 items divided into five categories: (a) appearance around the gallbladder, (b) appearance of Calot’s triangle, (c) appearance of the gallbladder bed, (d) additional findings related to the gallbladder and its surroundings, and (e) intra-abdominal findings unrelated to inflammation. The total difficulty score is calculated by summing the scores for all evaluated items.

Abbreviations: GB, gallbladder; TGDS18, surgical difficulty score from Tokyo guideline 2018

**Figure S4. Inter-rater agreement of surgical difficulty assessment between evaluators: The Bland-Altman plot for the total score**


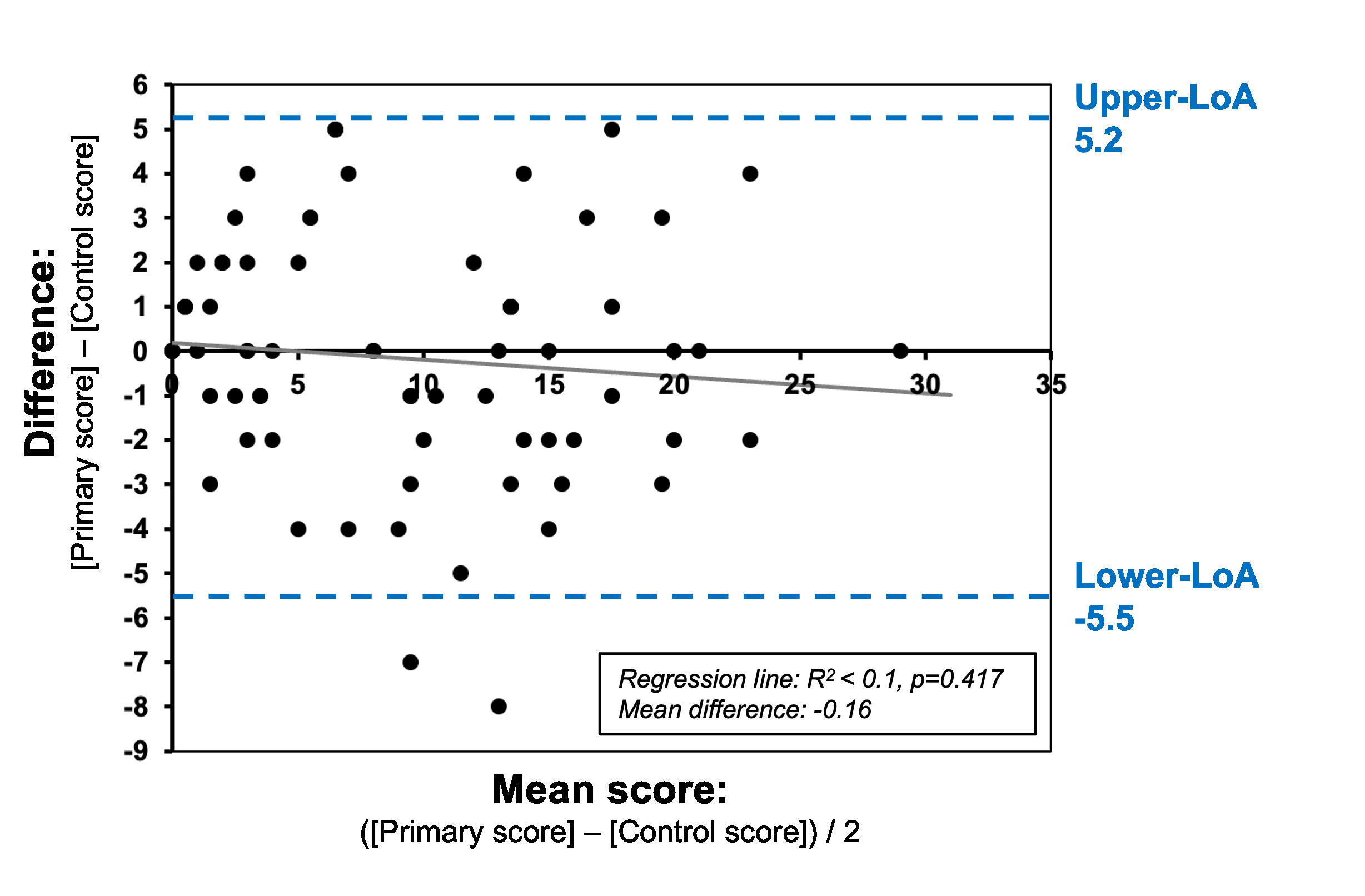


The agreement between the total score of TGDS18 used in this study (Primary Score) and the newly assessed score by another surgeon (Control Score) was visually evaluated using the Bland-Altman plot. The 95% limits of agreement (LoA) were calculated as the mean difference ± 1.96 standard deviations based on statistical standards. Most data points fell within this range, indicating a high level of agreement between the two scores. The results of the linear regression analysis (gray-colored line) showed R² < 0.1 (p = 0.417), statistically confirming the absence of bias. These findings provide statistical validation of the objectivity of TGDS18 used in this study.

Abbreviations: LoA, limits of agreement; TGDS18, surgical difficulty score from Tokyo guideline 2018

**Figure S5. Charlson comorbidity index and liver cirrhosis**


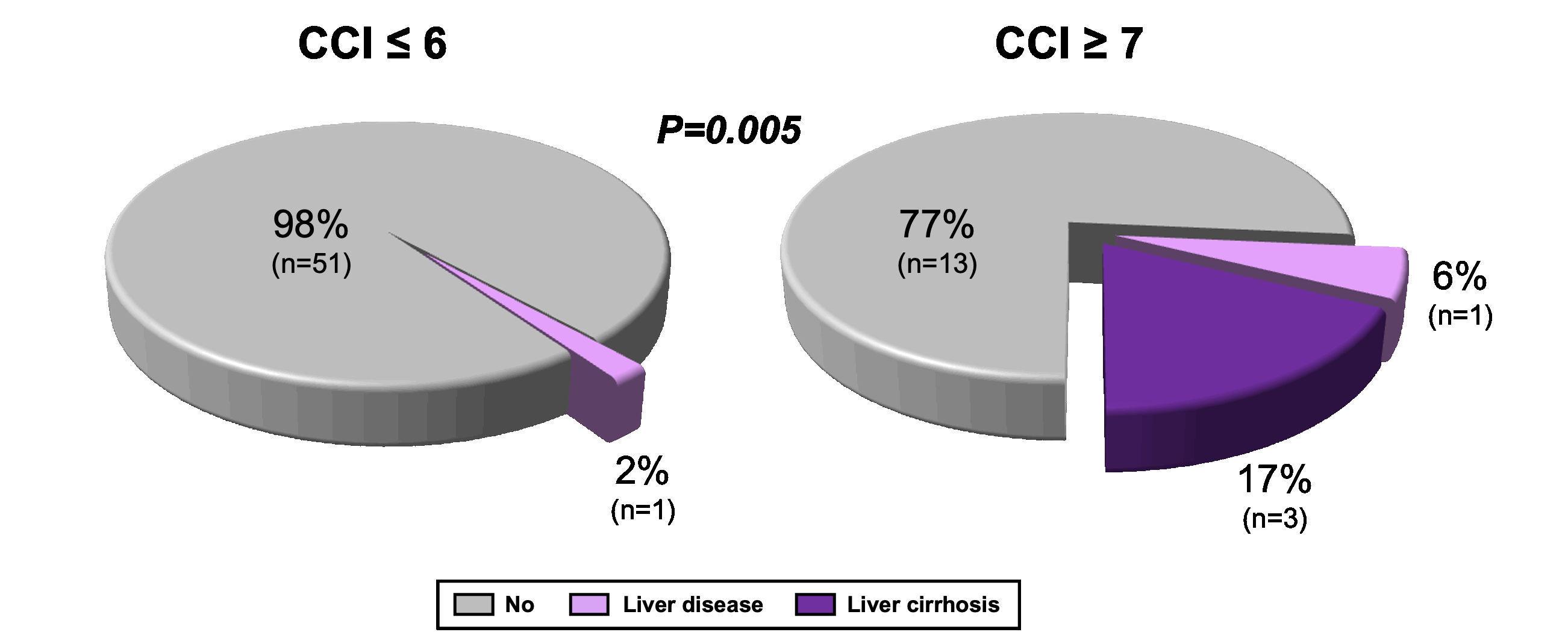


In a cohort of patients with pre-LC CT imaging (n=69), we assessed the confounding effects of the Age-adjusted Charlson comorbidity index (CCI) on liver cirrhosis. The proportions of patients with liver disease and cirrhosis were compared between the high CCI group (CCI ≥ 7) and the low CCI group (CCI ≤ 6). In the high CCI group, 17% had cirrhosis, while none in the low CCI group had cirrhosis (p=0.005).

Abbreviations: CCI, Age-adjusted Charlson comorbidity index
